# Genetic Diversity Analysis and Core Collection Construction of Ancient *Sophora japonica* L. Using SSR Markers

**DOI:** 10.3390/ijms252312776

**Published:** 2024-11-28

**Authors:** Yinyin Fu, Shuangyun Li, Bingyao Ma, Cuilan Liu, Yukun Qi, Caihong Pang

**Affiliations:** Key Laboratory of National Forestry and Grassland Administration on Tree Genetics and Breeding in the Yellow River Delta, Shandong Academy of Forestry, Jinan 250014, China; fuyin1029@163.com (Y.F.);

**Keywords:** ancient *S. japonica*, SSR, genetic diversity, genetic structure

## Abstract

*Sophora japonica* is an important native tree species in northern China, with high ornamental, medicinal, and ecological value. In order to elucidate the genetic resources of ancient *S. japonica*, 16 simple sequence repeat (SSR) markers were used to evaluate its genetic diversity and population structure and build a core collection of 416 germplasms from the Shandong, Shanxi, and Hebei provinces. A total of 160 alleles were detected, the mean major allele frequency (MAF)was 0.39, and the mean effective number of alleles (Ne) was 4.08. Shannon’s information index (I), the observed heterozygosity (Ho), the expected heterozygosity (He), and the polymorphism information content (PIC) were 1.58, 0.64, 0.74, and 0.70, respectively, indicating relatively high genetic diversity in ancient *S. japonica* germplasms. Low genetic differentiation coefficient (Fst = 0.04) and frequent gene flow (Nm = 9.74) were found in the tested *S. japonica* populations, and an analysis of molecular variance (AMOVA) indicated that the genetic variation mainly came from within individuals (84%). A genetic structure and cluster analysis indicated that 416 ancient *S. japonica* germplasms could be divided into five subgroups, and there were obvious genetic exchanges among different subgroups. A core collection consisting of 104 (25% of the original collection) germplasms was constructed using the R language package Genetic Subsetter version 0.8 based on the stepwise regression method. The retention rates of the number of alleles (Na), Ne, I, He, and PIC were 87.50%, 106.24%, 103.02%, 102.50%, and 102.74%, respectively. The *t*-test analysis suggested that there were no significant differences between the core collection and the original collection. The principal coordinate analysis (PCoA) showed that the core collection was uniformly distributed within the initial collection and was able to fully represent the genetic diversity of the original collection. These results provide a scientific basis for the conservation and utilization of ancient *S. japonica* germplasms.

## 1. Introduction

*Sophora japonica* L. is a deciduous tree belonging to the Papilionoideae family and is native to China and Korea, with extensive distribution across China [1]. *S. japonica* is tolerant to heat, salt, and drought, as well as severe frost, except during the seedling stage. The tree adapts to sandy, loamy, and clay soil well, and can even colonize on contaminated soil [2]. However, *S. japonica* grows best in well-drained and sandy loam. This species can tolerate atmospheric pollution and is intolerant to shade. The foliage of *S. japonica* is often described as luxurious, and its flowering occurs between July and August, when no other large trees are in bloom. The flower buds and fruits of *S. japonica* have been used in traditional Chinese medicine, as the bud and pericarp contain flavones, a hemostatic constituent [3]. In addition, the buds of *S. japonica* are used extensively in dyeing. The seeds are used in the treatment of hemorrhoids, hematuria, uterine bleeding, constipation, and hypertension [4]. With the above characteristics, *S. japonica* has become one of the hardiest ornamental and medicinal tree species.

During the investigation of germplasm resources, we found that there was no wild population of *S. japonica*. However, the number of ancient *S. japonica* trees (more than 300 years old) is huge, and they are found sporadically growing beside villages, on hillsides, roadsides, and watersides, and in parks and temples. These ancient trees have the characteristics of a long life and wide distribution. This was made possible by the genetic diversity of the ancient trees. Therefore, it is very important to investigate, collect, and preserve ancient *S. japonica* germplasm.

Molecular markers play important roles in plant molecular breeding, especially in the identification of genes responsible for desirable traits and genetic diversity. The most commonly used molecular markers are restriction fragment length polymorphism (RFLP), random amplified polymorphic DNA (RAPD), amplified fragment length polymorphism (AFLP), SSR, and single-nucleotide polymorphism (SNP). SSR markers have the advantage of high polymorphism, multi-allelic properties, co-dominance, specificity, extensive distribution, and relative stability, and have become the preferred method for the genetic mapping [5], germplasm identification [6], gene localization, molecular marker-assisted selection breeding, and genetic diversity analysis [7,8] of many plant species. In traditional SSR marker studies, polyacrylamide gel electrophoresis is commonly used. In recent years, capillaryrophoresis fluorescence labeling technology has gradually matured, and it is possible to distinguish between two gene fragments that differ by only 1 bp. This technology has played an important role in the large-scale identification of germplasm resources, especially those of forest trees [9,10,11,12].

In the 1980s, Frankel [13] proposed the concept of core collections. That is, the minimum number of germplasms represents the maximum genetic diversity. This concept can remove the germplasm materials with close genetic relationships, effectively alleviating the difficulties of large germplasm conservation. A core collection is an important part of artificial selective breeding and fine gene mining, and can improve the effective utilization rate of germplasm resources. At the same time, core collections help conserve unique and rare traits within a species. *S. japonica* is a perennial woody plant. The management of large germplasm resources is often costly and time-consuming. It is necessary to establish a core collection of *S. japonica*, reduce their management costs, and improve their utilization efficiency.

Currently, SSR molecular marker technology is mainly used for the genetic diversity analysis of *Sophora* species. Li et al. [14] analyzed the genetic diversity and genetic structure of *Sophora alopecuroides* populations using SSR molecular markers in combination with an association analysis between molecular markers and alkaloid contents. Research has shown that there has been higher genetic differentiation and less gene exchange among different *S. alopecuroides* populations. Shu et al. [15] analyzed the genetic diversity and population structure of 10 *S. japonica* populations using 26 SSR markers. The results revealed that there was a strong differentiation between most southern and northern populations. Lu et al. [16] developed 30 pairs of SSR primers based on transcriptomics and further used them to characterize the genetic diversity and genetic differentiation of five *S. japonica* populations. However, no research has been conducted on the genetic diversity of ancient *S. japonica*. Ancient *S. japonica* have survived in the natural environment for hundreds of years, indicating that they possess strong adaptability. Therefore, studying the diversity of ancient *S. japonica* resources is essential for breeding efforts and guiding conservation. In this study, the genetic diversity of 416 ancient *S. japonica* germplasm accessions was analyzed using SSR fluorescent labeling via capillary electrophoresis technology to elucidate the genetic structures and genetic backgrounds of germplasm resources. Additionally, a core collection was constructed and used as a genetic diversity library of germplasm resources, which could facilitate the breeding of new cultivars. This work offers a foundation for the breeding, preservation, and sustainable use of ancient *S. japonica* resources in China.

## 2. Results

### 2.1. SSR Markers and Their Allelic Diversity

A total of 160 clear bands were amplified, with an average of 10 alleles per SSR locus (Table 1). A maximum of 19 alleles were revealed by Locus 2844 and a minimum of 4 alleles were revealed by Locus 2541. The Ne per locus varied from 2.55 to 5.99 (mean: 4.08). The major allele frequency ranged from 0.25 (Locus 2270) to 0.58 (Locus 1820) with an average of 0.39. All SSR markers were found to be highly informative, with a PIC value of ≥0.50. Loci 2128 and 2844 showed the highest PIC (0.81), while Locus 1820 showed the lowest PIC (0.57).

### 2.2. Genetic Diversity Analysis

In this study, high genetic diversity was shown among the 416 germplasm resources. The genetic variation was estimated using the Ho, He, I, Fis, Fst, and the total population inbreeding coefficient (Fit) (Table 1). The results of I ranged from 1.21 (Locus 1820) to 2.12 (Locus 2844), with an average of 1.58. A maximum He value of 0.83 (for 2128 and 2844), a minimum value of 0.61 (for 1820), and an average of 0.74 for all loci values were observed. The values of Ho ranged from 0.33 for Locus 756 to 0.81 for Locus 2128, with a mean value of 0.64. The Fis varied from −0.07 to 0.33, and negative values were observed at six loci, including 2434, 2128, 2114, 1970, 1820, and 1527. Out of all loci, only 1609, 2114, and 2434 showed moderate degrees of genotypic differentiation among the populations, with Fst values of more than 0.05. The Nm ranged from 2.12 to 23.20, with a mean value of 9.74.

In addition, across the population, high (0.737) and low (0.663) values of He were observed in Pop SD and SX. The values of Ho ranged from 0.637 to 0.678. The average NA, NE, and I were 7.08, 3.647, and 1.428, respectively (Table 2). The results showed that the genetic diversity of the three Pop was high.

AMOVA using GenAlEx version 6.503 revealed a genetic variation of 84% within individuals. The remaining 13% existed among individuals, and 3% was present among the Pops (Table 3).

### 2.3. Clustering and Population Genetic Structure

**Figure 1:**
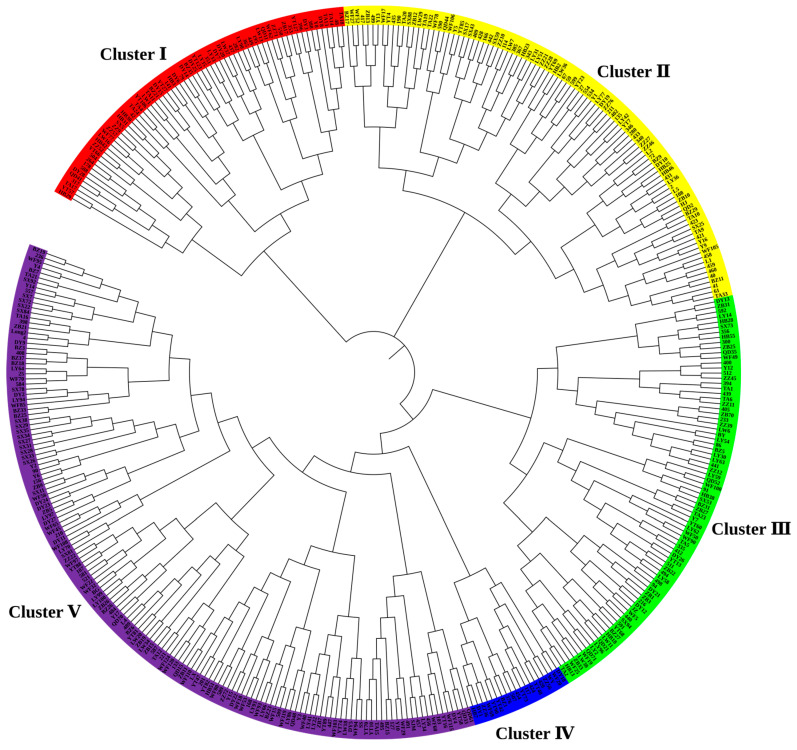
UPGMA clustering analysis of 416 ancient *S. japonica* germplasm resources.

Based on Nei’s genetic distance method, we constructed a phylogenetic tree of 416 *S. japonica* germplasms, and five clusters were obtained (Figure 1). There were 66, 98, 83, 19, and 150 germplasm resources in Cluster I, Cluster II, Cluster III, Cluster IV, and Cluster V, respectively. Cluster IV consisted of 19 germplasms—18 from Pop SD and 1 from Pop HB. The other four clusters’ germplasms were from Pop SD, HB, and SX (Table S1). The clustering analysis indicated that the germplasms from different geographical sources formed distinct clusters, and geographic isolation did not significantly affect the genetic differentiation of *S. japonica*.

**Figure 2:**
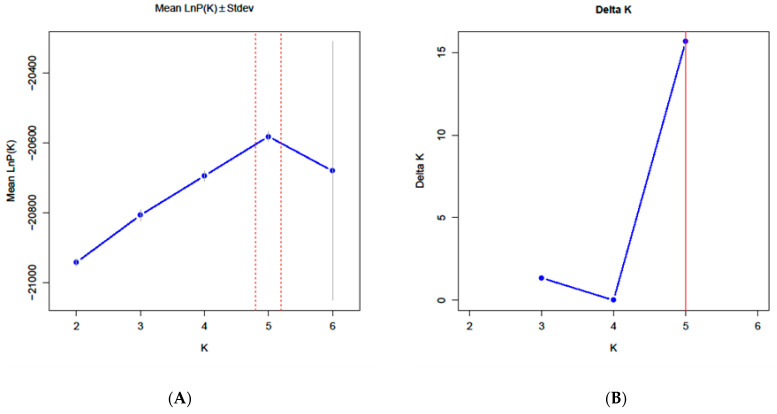
The optimal population number of *S*. *japonica* field populations detected using Structure software (K). (**A**) The average Lnp (D) value of each K value based on 10 repetitions; (**B**) the K value plot as it changed with ΔK.

**Figure 3:**
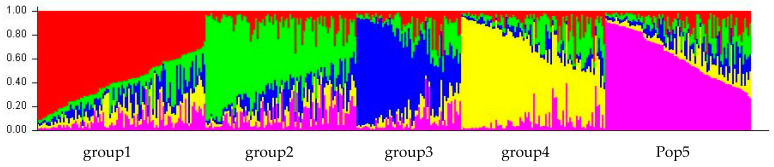
Population structure of 416 ancient *S. japonica* germplasms based on 16 SSR markers at k = 5.

Structure software version 2.3.4 was used to analyze the population genetic structure of the 416 germplasm resources. The default K value was set from 2 to 6 and repeated 10 times. The number of Markov Chain Monte Carlo (MCMC) iterations was set to 100,000 for each run. The optimal value of K was calculated to be 5 (Figure 2). Therefore, all the *S. japonica* germplasms were divided into five groups (Figure 3, Table S4). Group 1 included 85 germplasms—83 germplasms from Pop SD and 2 from Pop HB. Group 2 contained 83 germplasms—53 from Pop SD, 25 from Pop SX, and 5 from Pop HB. Group 3 included 67 germplasms—64 from Pop SD, 1 from Pop SX, and 3 from Pop HB. Group 4 included 85 germplasms—80 from Pop SD and 5 from Pop HB. Group 5 included 96 germplasms—95 from Pop SD and 1 from Pop HB. The expected heterozygosity was analyzed for five suspected groups (Table S2). The highest He was shown in group 4 with 0.725, followed by group 3 with 0.716. The lowest He was observed in group 5 with 0.705.

### 2.4. Selection of the Core Collection

**Figure 4:**
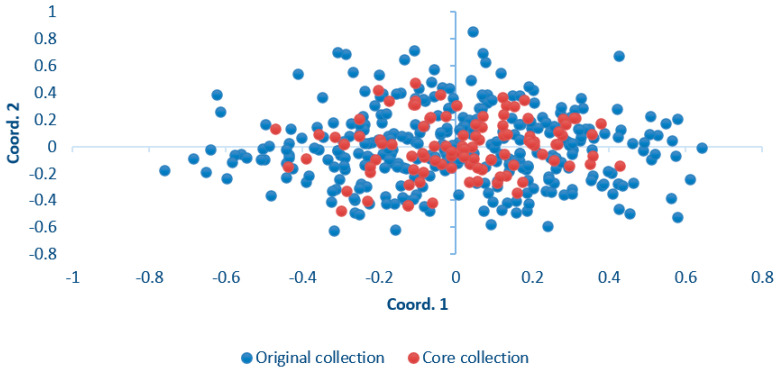
The principal coordinate distribution of the core collection and the original collection of ancient *S. japonica.*

Ten candidate core collections of ancient *S. japonica* were constructed using the R package Genetic Subsetter version 0.8, including sampling ratios of 50% (208), 45% (187), 40% (166), 35% (146), 30% (125), 25% (104), 20% (83), 15% (62), 10% (42), and 5% (21). The genetic diversity parameters of the Na, Ne, He, I, and PIC were used to analyze the candidate core collection (Table S3). The results show that the Na decreased, whereas the Ne, He, I, and PIC increased with decreases in the sampling proportion. The Ne, I, and PIC reached their maximum values when the sampling ratio was 25%, and the He reached its maximum value when the sampling ratio was 20%. Therefore, a total of 104 collections were generated from population SD, HB, and SX, forming a core collection that accounted for 25% of the original collections in this study. The retention rates of the Na, Ne, I, He, and PIC of these 104 core collections were 87.50%, 106.24%, 103.02%, 102.50%, and 102.74%, respectively. Except for the Na, the parameters were higher than those of the original and reserved collection (Table 4). There was no significant difference between the genetic diversity index of the core collection and the original collection using the *t*-test (Table 4). The core collection was further confirmed using principal coordinate analysis (PCoA). The results show that the 104 core collections were evenly distributed in the principal coordinate map of the original collection (Figure 4).

## 3. Discussion

Independent of environmental and genotype variations, molecular markers can facilitate breeding through marker-assisted selection (MAS) [17,18]. SSR markers have already been successfully applied to the evaluation of the genetic diversity, population structure, and core collection construction of forest trees [19,20,21,22,23]. In this study, effective SSRs were developed for the identification and classification of ancient *S. japonica*. There were more than four alleles at the 16 SSR loci that were used for further genetic diversity analysis of ancient *S. japonica*. A total of 160 clear bands were amplified in 416 different *S. japonica* germplasms, and all of them were polymorphic (100%). The PIC values varied from 0.57 to 0.81, and the average value was 0.70. Based on the PIC criteria [24], the markers developed in this study provide a valuable reference for genetic research of ancient *S. japonica*. High genetic diversity may lead to the strong adaptability of plants to environmental changes [25]. The mean Ne, I, and He values were 4.08, 1.58, and 0.74, respectively. Therefore, these 416 germplasms had relatively high genetic diversity. These results are in agreement with the previous results of Shu et al. [15] and higher than those of Lu et al. [16]. This may be due to the source and quantity of the germplasm resources.

The Fis values of all germplasms were 0.05, indicating that the probability of inbreeding was low in ancient *S. japonica* (Table 1). This may be another reason for the high genetic diversity of ancient *S japonica*. Of all the tested sites, the He was higher than the Ho, which indicates that heterozygote deletion existed to different degrees. Meanwhile, the results found in *S. japonica* (He < Ho) [15] were the opposite, perhaps because their samples were collected from semi-wild populations and landrace populations.

Generally, populations show moderate genetic differentiation when the Fst is 0.05~0.15 and low genetic differentiation when the Fst is <0.05 [26,27]. This study shows that ancient *S. japonica* had a low level of genetic differentiation (Fst = 0.04), and most of the genetic variation came from within the population. The AMOVA also revealed that 84% of the genetic variation of *S. japonica* germplasms was within individuals (Table 3). These results are consistent with those found in an *S. japonica* population in a previous study [15]. Wright [28] suggested that there was a certain gene flow that could prevent genetic differentiation between families caused by genetic drift when Nm > 1; and strong genetic differentiation occurred in the population when Nm < 1. The Nm of ancient *S. japonica* ranged from 2.12 to 23.20, with a mean value of 9.74 (Table 1). Higher levels of gene flow can attenuate the effects of genetic drift on a population’s genetic structure, which may be one of the reasons for the lower level of genetic differentiation in the tested germplasms. The reproduction of *S. japonica* mainly depends on the seed, which may be the main reason for gene flow.

In this study, an unweighted pair group method with an arithmetic (UPGMA) clustering analysis provided clear evidence for the genetic relationships among 416 germplasms. The dendrogram generated five major clusters. Cluster IV consisted of 19 germplasms—18 from Pop SD and 1 from Pop HB. The other four clusters’ germplasms were from Pop SD, HB, and SX. These results suggest that there was no clear separation among the genotypes according to their geographical locations. The results obtained from the structure analysis are almost in accordance with those of the UPGMA, inferring that 416 germplasms were clustered into five groups. These results indicate that *S. japonica* variants have a common genetic background and common alleles among them.

The selection of a core collection is an effective way to facilitate the efficient, scientific, and rational conservation and utilization of genetic diversity. A reasonable sampling proportion is a critical factor for constructing a core collection. Brown [29] suggested that 5–10% of a core collection could represent more than 70% of the genetic variation of all germplasm resources. The sampling rate of different plants for a core collection is usually 5–40%. The present research shows that the sampling proportions of core collections of forest tree germplasm resources are generally 10–30% [30,31,32,33,34]. It is generally accepted that the sampling proportion can be appropriately reduced for species with large original germplasms and low genetic diversity. On the contrary, it is necessary to increase the sampling proportion for species with small original germplasms and high genetic diversity [35]. In this study, 104 core accessions were selected from 416 original accessions based on the stepwise regression method according to the results of the SSR marker analysis, and the suitable sampling rate of the core collection was 25%. The retention rates of the Na, Ne, I, He, and PIC of the 104 core collections were 87.50%, 106.24%, 103.02%, 102.50%, and 102.74%, respectively. This indicates that the core collection effectively eliminated the genetic redundancy of the original collection and retained more genetic variation. The PCoA and *t*-test showed that the core collection could reflect the distribution of the original collection. The core collection constructed in this study could represent the genetic diversity of the original germplasms. To a certain degree, the analysis of genetic diversity and the construction of core collections are helpful for the selective conservation of ancient *S. japonica*, reducing their management costs and improving their utilization efficiency. The extent to which genetic diversity is represented is an important indicator of the effectiveness of a core collection. However, a core collection cannot fully encompass the entire genetic diversity of a species. An effective solution is to allow for the size and composition of the core collection to evolve over time. In order to maintain the genetic diversity of *S. japonica*, it is necessary to continuously incorporate new germplasm resources into the core collection. This study is a preliminary attempt to establish a core collection for *S. japonica*. Given the limited number of markers and the lack of phenotypic data, future studies should aim to complement and refine these findings.

At present, the breeding of *S. japonica* is carried out through the selection of superior trees, and the objectives during ex situ conservation are diverse. Directional hybrid breeding is the general current trend. The genetic diversity and structure analysis showed that the genetic background of *S. japonica* resources from the same provenance might differ. *S. japonica* resources from different provenances were also clustered into the same groups. Before hybrid breeding, understanding the genetic background and carrying out targeted germplasm innovation could greatly improve the breeding efficiency of *S. japonica*. It is essential to determine the parents in advance according to the breeding objectives.

## 4. Materials and Methods

### 4.1. Plant Materials

A total of 416 ancient *S. japonica* (more than 300 years old) were collected from the Shandong, Hebei, and Shanxi provinces. They had been preserved for more than 10 years at the *S. japonica* germplasm nursery in Jinan City, Shandong province (36°25′–37°09′ N, 117°10′–117°35′ E, with an average annual temperature of approximately 12.8 °C and average annual precipitation of 600 mm) (Table 5). Tender leaves of 10 individuals were sampled and pooled for each germplasm, and then stored at −80 °C.

### 4.2. Genomic DNA Extraction and PCR Amplification

The genomic DNA of each germplasm was extracted from the pooled fresh leaves using a Plant Genomic DNA Kit (Tiangen, Beijing, China). SSR primer pairs were randomly developed in our laboratory from the transcriptome sequences of *S. japonica* with the following configurations: containing different di/tri-nucleotide repeats; a motif product size of 100 to 300 bp; a primer length of 18 to 28 bp; a GC percentage of 40 to 60%; and an annealing temperature varying between 55 and 65 °C. All 90 primer pairs were screened, with 8 individuals randomly sampled to detect their polymorphisms (Figure S1). Finally, we selected 16 primer pairs that could provide reproducible and polymorphic DNA amplification products with an expected size. The information on the 16 primers marked with four different fluorescences is given in Table 6. PCR amplification was performed in a volume of 10 μL: 0.05 μL of ExTaq enzyme (TaKaRa, 5 U/L), 1.0 μL of Mg^2+^ (25 mmol/L), 0.5 μL of primer (10 mmol/L), 1.0 μL of template DNA (30 ng/μL), 0.2 μL of dNTPs (2.5 mmol/L), 1 μL of 10 × Taq Buffer, and 6.45 μL of ddH2O. A touchdown PCR amplification procedure was performed as follows: denaturation at 94 °C for 5 min, followed by 17 cycles of 15 s at 94 °C, 15 s at Tm (annealing temperature of 66.5 °C–57 °C, at steps of −0.5 °C per cycle), 30 s at 72 °C, 15 cycles of 15 s at 94 °C, 15 s at 57 °C, 30 s at 72 °C, and a final step at 72 °C for 10 min. The PCR products were identified via capillary electrophoresis using an ABI 3730XL sequencer (Applied Biosystems, Carlsbad, CA, USA). The alleles were read using the GeneMarker v 2.2.0 software package (SoftGenetics LLC, State College, PA, USA).

### 4.3. Genetic Diversity and Population Structure Analysis

The genetic diversity parameters were calculated with the GenAlEx version 6.503 software [36,37], including the Na, Ne, He, Ho, I, Fis, Fit, and the Nm. Pairwise FST values and AMOVA were also carried out. The MAF and the PIC of each pair of SSR primers were calculated with PowerMarker version 3.25. According to Nei’s genetic distance, a phylogenetic tree was constructed using an UPGMA mean with PowerMarker version 3.25. The population structures were analyzed using STRUCTURE version 2.3.4 software [38], which was run for 10 iterations following a burn-in period of 10,000 iterations with K values of 2–6, using the admixture model.

### 4.4. Development of the Core Collection

The core collection was constructed using the R language package Genetic Subsetter version 0.8 based on the stepwise regression method [39,40,41]. All the samples were sorted according to their general contribution to the mean expected heterozygosity. In a stepwise process, the samples with the lowest contribution to the He were removed, and the average He was recalculated. A subset that represents the allelic diversity of the whole collection was finally established. The Na, Ne, I, He, and PIC values were calculated for eight newly sorted samples: 50%, 45%, 40%, 35%, 30%, 25%, 20%, 15%, 10%, and 5%. SPSS v20.0 (IBM SPSS Statistics version 22.0; IBM Corp., Armonk, NY, USA) was used to evaluate the final core collection by performing *t*-test for the Na, Ne, I, He, and PIC values between the core and original collections.

## Figures and Tables

**Table 1:** Genetic diversity of 416 different ancient *S. japonica* germplasms based on SSR markers.

SSR	MAF	N	Na	Ne	I	Ho	He	Fis	Fit	Fst	Nm	PIC
2541	0.41	416	4	3.32	1.28	0.65	0.70	0.09	0.10	0.01	23.20	0.64
2128	0.28	416	10	5.99	1.95	0.81	0.83	−0.06	−0.02	0.04	6.35	0.81
2114	0.40	415	7	3.66	1.50	0.66	0.73	−0.04	0.02	0.06	3.93	0.69
1970	0.30	410	10	5.35	1.84	0.77	0.81	−0.02	0.00	0.02	10.66	0.79
2844	0.33	416	19	5.90	2.12	0.57	0.83	0.20	0.23	0.04	6.27	0.81
3049	0.36	415	13	4.19	1.71	0.68	0.76	0.01	0.04	0.03	7.45	0.73
1609	0.54	412	8	2.76	1.25	0.54	0.64	0.15	0.21	0.07	3.48	0.59
1302	0.56	416	10	2.79	1.41	0.49	0.64	0.10	0.13	0.04	6.78	0.61
1060	0.33	416	15	4.00	1.66	0.71	0.75	0.03	0.04	0.01	18.33	0.71
2434	0.34	416	7	3.60	1.42	0.66	0.72	−0.04	0.07	0.11	2.12	0.67
1820	0.58	416	7	2.55	1.21	0.58	0.61	−0.01	0.02	0.03	8.40	0.57
1663	0.38	414	8	3.20	1.28	0.62	0.69	0.04	0.05	0.02	13.97	0.63
953	0.37	416	11	4.76	1.83	0.76	0.79	0.03	0.06	0.03	8.98	0.77
1527	0.47	416	6	3.03	1.25	0.62	0.67	−0.07	−0.06	0.02	15.04	0.62
2270	0.25	414	12	5.24	1.79	0.79	0.81	0.04	0.07	0.03	7.30	0.78
756	0.31	412	13	5.00	1.80	0.33	0.80	0.33	0.34	0.02	13.59	0.77
Mean	0.39		10	4.08	1.58	0.64	0.74	0.05	0.08	0.04	9.74	0.70

Abbreviations: MAF, major allele frequency; N, number of samples; Na, number of alleles; Ne, effective number of alleles; I, Shannon’s information index; Ho, observed heterozygosity; He, expected heterozygosity; Fis, intrapopulation inbreeding coefficient; Fit, total population inbreeding coefficient; Fst, genetic differentiation coefficient; Nm, gene flow; PIC, polymorphism information content.

**Table 2:** Genetic diversity of three ancient *S. japonica* Pops.

Pop	N	Mean Na	Mean Ne	I	Ho	He
SD	375	9.625	4.085	1.575	0.637	0.737
HB	16	5.313	3.538	1.353	0.678	0.678
SX	26	6.313	3.319	1.357	0.662	0.663
Total		7.083	3.647	1.428	0.659	0.693

**Table 3:** Molecular variance analysis of ancient *S. japonica*.

Source	df	SS	MS	Est. Var.	%
Among Pops.	2	42.582	21.291	0.189	3
Among Indiv.	413	2742.699	6.641	0.766	13
Within Indiv.	416	2125.000	5.108	5.108	84
Total	831	4910.281		6.064	100

Abbreviations: df, degrees of freedom; SS, sum of squares; MS, mean of the squares; Est. Var., estimated variance of components; %, percentage of total variance contributed by each component.

**Table 4:** A comparison of the genetic diversity of the original, core, and reserved collections; a *t* test of the genetic diversity parameters between the core collection and the original collection.

Germplasm	N	Na	Ne	I	Ho	He	Fst	PIC
Original collection	416	10.00	4.08	1.58	0.64	0.74	0.04	0.70
Core collection	104	8.75	4.34	1.63	0.78	0.76	0.05	0.72
Retention rate (%)	25.00	87.50	106.27	103.04	122.05	102.55	141.95	102.74
Retention collection	312	6.42	3.50	1.37	0.59	0.67	0.05	0.69
Retention rate (%)	75.00	64.17	85.73	86.58	91.69	91.39	135.07	98.78
*t*-test		0.31	0.54	0.64	0.00	0.44	0.12	0.49

**Table 5:** Source and number of ancient *S. japonica* germplasms.

Abbreviation	Source	Number	Latitude	Longitude
SD	Shandong Provinces	374	34°22′~38°23′ N	114°19′~122°43′ E
HB	Hebei Provinces	16	36°05′~42°40′ N	113°27′~119°50′ E
SX	Shanxi Provinces	26	34°36′~40°44′ N	110°15′~114°32′ E

**Table 6:** Characteristics of 16 microsatellite primers for ancient *S. japonica.*

Primer Name	Repeated Motif	Primer Sequences (5′-3′)	Size Range (bp)	Annealing Temperature (°C)
2541	(TG) 9	F: AATGTTGAATGGAATTTGGACAC R: TCTCTCTGAATATCTCTTCCCCC	146–156	57
1302	(AAG) 6	F: ATTCGGAAGAGGTTGTTGACAT R: TCCCTTCGATCTCTTTCTTCTCT	118–134	57
2128	(TG) 10	F: CCTCCTTGTAGTAGCCACAACTG R: AGACAATCATAAGCACCGTCTTC	122–140	57
1609	(AC) 7	F: CAACCCTAGAGTGTCTCCTTGAA R: TCAAAGGAACCAAAGAAACAAAA	130–148	57
2844	(TA) 8	F: CACGACATTTCAATGTGTACTGC R: CTGAAGCAATGCAAAATCATACC	100–132	57
3049	(GA) 8	F: ACCTTTCACTCAGCTGACACAGT R: AAAAGGAACGAGAAAACCAAAAT	158–188	57
1970	(ACA) 7	F: AAACCCTCAGGACTTCTCACAAC R: AAAGTCCTTCACGAAGACGAAAC	72–102	57
2114	(GAA) 7	F: GGTTCAGAATCAGAAGCAAAAGA R: GAGTGGAAAACAGAGTGGAGAGA	147–165	57
1060	(TGT) 6	F: GTAACATTCCACCGAACCCATA R: ATCGAGGGTTTCTTCAAAGTTTC	97–131	57
2434	(AC) 8	F: AAAAGAATAGAATGGAAGGCCAG R: GCATAATTTGAACAAGGCACAGT	143–155	57
1663	(GA) 8	F: TTATTTCTAGCGAAATCGGACAC R: TCGCTCACTTACTCACACTCAAA	121–129	57
1820	(AC) 8	F: GGCCTTGTTAGGAATTTGTCTTT R: AAAACATAAGTGCCAATTCATCC	107–117	57
953	(AG) 8	F: CTTCCATTTTCCCTCACTTTCTC R: ACATCGTTCCTCACGTCGTAGT	148–174	57
1527	(GA) 8	F: AATCTAAAGCCAGCTCAACGAG R: AGTAATGGTGGTGGACAGAGAAA	160–168	57
2270	(AG) 7	F: CTCCATGGTTGAGGAGTTTGTTA R: CAGTCCAAGAAAAGGACAAGAAA	159–183	57
756	(TA) 8	F: TTCCTCTCCTTACAGTGCCATTA R: GCAGTTTATGTGACTTGAGGTCC	118–146	57

## Data Availability

Data is contained within the article and Supplementary Materials.

## Supplementary Materials

The supporting information can be downloaded at https://www.mdpi.com/article/10.3390/ijms252312776/s1.
