# Supplementary material for: Genetic Diversity Analysis and Core Collection Construction of Ancient Sophora japonica L. Using SSR Markers
Source: Int J Mol Sci. 2024 Nov 28;25(23):12776. doi: 10.3390/ijms252312776 (PMC11641213; doi:10.3390/ijms252312776)

Table S1 Germplasm number of three populations in each of the five inferred cluster by the UPGMA clustering analysis

Inferred clusters	SD	HB	SX	The total number ofcluster
cluster 1	60	5	1	66
cluster 2	89	4	5	98
cluster 3	75	5	3	83
cluster 4	18	1	0	19
cluster 5	132	1	17	150
The total number of	374	16	26	416

Table S2. Germplasm number of three populations in each of the five inferred groups.

Inferred clusters	Germplasm number			Total number of inferred clusters	Average Q value	He
	SD	HB	SX			
group1	83	2	0	85	0.612	0.707
group2	53	5	25	83	0.646	0.709
group3	64	3	1	67	0.595	0.716
group4	80	5	0	85	0.562	0.725
group5	95	1	0	96	0.595	0.705
Total	375	16	26	416	0.602	0.736

Table S3. Comparison of genetic diversity under various sampling ratios.

Sample number	Sampling ratio (%)	Na	Ne	I	He	PIC
416	100	10.00	4.08	1.58	0.74	0.700
208	50	9.38	4.23	1.61	0.75	0.711
187	45	9.19	4.25	1.61	0.75	0.712
166	40	9.19	4.24	1.61	0.75	0.711
146	35	8.94	4.25	1.61	0.75	0.713
125	30	8.75	4.31	1.62	0.75	0.716
104	25	8.75	4.34	1.63	0.75	0.719
83	20	8.69	4.33	1.63	0.76	0.719
62	15	5.56	3.57	1.36	0.68	0.718
42	10	5.08	3.53	1.32	0.68	0.715
21	5	4.48	3.44	1.29	0.68	0.708

Table S4 Sample distribution of each group when K=5

POP	sample	Q value				
		group 1	group 2	group 3	group 4	group 5
SD	2	0.09	0.188	0.455	0.229	0.037
	4	0.032	0.504	0.026	0.397	0.042

25	0.019	0.04	0.019	0.025	0.898
27	0.056	0.06	0.299	0.079	0.506
40	0.032	0.075	0.784	0.02	0.089
41	0.016	0.024	0.035	0.089	0.836
42	0.04	0.106	0.385	0.3	0.169
48	0.044	0.036	0.472	0.108	0.339
59	0.034	0.503	0.306	0.116	0.041
61	0.21	0.026	0.161	0.028	0.574
67	0.061	0.04	0.15	0.217	0.532
72	0.097	0.038	0.123	0.033	0.709
79	0.111	0.636	0.02	0.195	0.038
86	0.044	0.05	0.382	0.481	0.042
90	0.5	0.363	0.038	0.031	0.069
91	0.127	0.061	0.756	0.03	0.025
94	0.072	0.277	0.062	0.491	0.098
103	0.04	0.718	0.093	0.066	0.083
107	0.02	0.048	0.503	0.368	0.061
108	0.014	0.026	0.671	0.243	0.046
114	0.16	0.664	0.06	0.051	0.065
117	0.055	0.059	0.063	0.051	0.772
127	0.022	0.495	0.094	0.222	0.167
133	0.024	0.523	0.028	0.392	0.034
135	0.284	0.032	0.231	0.06	0.393
141	0.327	0.054	0.275	0.274	0.069
156	0.037	0.744	0.102	0.083	0.035
166	0.126	0.449	0.034	0.342	0.048
168	0.337	0.184	0.113	0.218	0.148
178	0.384	0.187	0.156	0.217	0.055
190	0.517	0.054	0.178	0.139	0.112
195	0.024	0.256	0.586	0.04	0.093
201	0.07	0.138	0.352	0.046	0.394
210	0.044	0.024	0.043	0.069	0.819
211	0.493	0.199	0.11	0.086	0.112
216	0.064	0.305	0.034	0.417	0.179
221	0.049	0.083	0.522	0.294	0.051
233	0.138	0.066	0.155	0.616	0.026
236	0.025	0.568	0.23	0.043	0.134
264	0.348	0.31	0.138	0.176	0.028
280	0.118	0.015	0.762	0.048	0.057
282	0.186	0.198	0.221	0.038	0.357
300	0.307	0.086	0.197	0.374	0.037
317	0.388	0.114	0.045	0.419	0.034
335	0.045	0.016	0.873	0.051	0.015
340	0.84	0.032	0.042	0.052	0.033

343	0.519	0.145	0.038	0.089	0.209
346	0.129	0.042	0.64	0.025	0.164
351	0.386	0.12	0.047	0.08	0.366
353	0.245	0.136	0.309	0.221	0.089
356	0.033	0.024	0.89	0.036	0.016
365	0.06	0.033	0.083	0.064	0.76
367	0.35	0.409	0.072	0.098	0.07
376	0.826	0.051	0.025	0.04	0.057
385	0.635	0.245	0.055	0.045	0.02
388	0.655	0.034	0.072	0.054	0.186
394	0.443	0.019	0.251	0.086	0.201
396	0.455	0.025	0.072	0.069	0.379
398	0.077	0.096	0.036	0.039	0.752
400	0.851	0.038	0.045	0.041	0.026
402	0.518	0.044	0.279	0.042	0.117
405	0.382	0.239	0.033	0.135	0.211
408	0.13	0.031	0.192	0.048	0.598
409	0.072	0.493	0.035	0.171	0.229
412	0.114	0.017	0.819	0.027	0.022
417	0.753	0.053	0.08	0.082	0.032
420	0.567	0.032	0.128	0.065	0.207
421	0.623	0.149	0.046	0.117	0.065
423	0.363	0.513	0.026	0.055	0.042
424	0.649	0.03	0.192	0.046	0.083
428	0.231	0.151	0.262	0.145	0.212
431	0.102	0.211	0.518	0.059	0.109
433	0.022	0.017	0.916	0.018	0.028
435	0.687	0.028	0.035	0.069	0.181
437	0.305	0.207	0.137	0.243	0.108
439	0.89	0.014	0.045	0.033	0.018
440	0.149	0.529	0.093	0.153	0.076
441	0.332	0.054	0.053	0.079	0.482
442	0.467	0.163	0.028	0.319	0.025
447	0.251	0.019	0.449	0.054	0.228
449	0.088	0.257	0.05	0.037	0.568
454	0.437	0.189	0.126	0.226	0.022
456	0.871	0.022	0.039	0.033	0.035
457	0.721	0.052	0.029	0.065	0.133
458	0.878	0.028	0.024	0.053	0.017
459	0.701	0.016	0.172	0.042	0.068
460	0.788	0.02	0.093	0.059	0.039
484	0.08	0.107	0.063	0.1	0.65
489	0.084	0.066	0.083	0.674	0.093
491	0.051	0.114	0.059	0.19	0.585

494	0.033	0.017	0.874	0.037	0.04
495	0.018	0.019	0.041	0.053	0.869
498	0.031	0.023	0.036	0.865	0.044
499	0.093	0.044	0.067	0.093	0.703
504	0.1	0.112	0.37	0.227	0.192
507	0.388	0.055	0.066	0.417	0.074
509	0.03	0.029	0.034	0.067	0.84
511	0.023	0.041	0.811	0.02	0.106
512	0.37	0.022	0.162	0.401	0.044
521	0.212	0.064	0.066	0.56	0.099
534	0.582	0.236	0.035	0.042	0.105
550	0.536	0.034	0.065	0.122	0.243
554	0.349	0.039	0.288	0.263	0.06
557	0.039	0.819	0.026	0.026	0.09
581	0.029	0.038	0.028	0.822	0.083
584	0.052	0.197	0.155	0.052	0.543
587	0.146	0.03	0.032	0.756	0.036
588	0.363	0.047	0.385	0.142	0.063
589	0.189	0.204	0.298	0.243	0.067
590	0.182	0.231	0.092	0.372	0.123
591	0.204	0.063	0.058	0.491	0.185
592	0.136	0.019	0.736	0.089	0.02
599	0.034	0.171	0.62	0.044	0.131
BG	0.024	0.218	0.019	0.715	0.024
BY	0.043	0.026	0.349	0.267	0.315
BZ1	0.175	0.227	0.062	0.226	0.31
BZ11	0.029	0.023	0.751	0.161	0.036
BZ15	0.017	0.065	0.114	0.106	0.698
BZ17	0.029	0.027	0.038	0.862	0.044
BZ18	0.07	0.071	0.376	0.123	0.36
BZ19	0.028	0.298	0.065	0.053	0.556
BZ21	0.058	0.451	0.098	0.341	0.052
BZ23	0.018	0.026	0.062	0.165	0.729
BZ25	0.02	0.359	0.065	0.278	0.279
BZ26	0.032	0.54	0.026	0.167	0.235
BZ27	0.042	0.023	0.047	0.109	0.779
BZ29	0.12	0.473	0.064	0.111	0.232
BZ3	0.149	0.073	0.167	0.049	0.561
BZ31	0.024	0.031	0.878	0.022	0.045
BZ33	0.026	0.069	0.519	0.081	0.306
BZ35	0.092	0.283	0.116	0.059	0.45
BZ37	0.037	0.024	0.087	0.07	0.783
BZ5	0.07	0.27	0.063	0.22	0.376
BZ6	0.033	0.491	0.113	0.261	0.102

BZ7	0.027	0.501	0.03	0.025	0.417
BZ9	0.139	0.057	0.108	0.041	0.655
DY1	0.16	0.229	0.053	0.422	0.137
DY10	0.057	0.601	0.078	0.205	0.058
DY11	0.083	0.092	0.053	0.111	0.66
DY12	0.134	0.093	0.258	0.319	0.195
DY13	0.036	0.059	0.49	0.373	0.042
DY14	0.024	0.242	0.168	0.087	0.479
DY15	0.025	0.459	0.022	0.027	0.466
DY18	0.248	0.046	0.356	0.238	0.111
DY19	0.241	0.208	0.09	0.4	0.061
DY2	0.025	0.043	0.023	0.021	0.887
DY20	0.028	0.319	0.37	0.235	0.046
DY21	0.028	0.052	0.04	0.619	0.261
DY22	0.212	0.098	0.096	0.412	0.183
DY23	0.026	0.899	0.013	0.04	0.022
DY24	0.017	0.845	0.019	0.038	0.08
DY25	0.719	0.085	0.031	0.1	0.065
DY26	0.027	0.104	0.808	0.023	0.038
DY27	0.27	0.532	0.033	0.124	0.041
DY28	0.041	0.408	0.169	0.332	0.05
DY3	0.108	0.112	0.154	0.226	0.399
DY4	0.024	0.04	0.084	0.673	0.18
DY5	0.301	0.058	0.213	0.365	0.064
DY6	0.064	0.06	0.067	0.117	0.692
DY7	0.036	0.439	0.054	0.054	0.418
DY8	0.066	0.063	0.185	0.594	0.093
DY9	0.026	0.611	0.064	0.228	0.072
HJ	0.185	0.071	0.492	0.081	0.171
JZ	0.442	0.051	0.239	0.2	0.068
L1	0.877	0.026	0.035	0.041	0.021
L2	0.347	0.032	0.079	0.124	0.418
L4	0.268	0.094	0.042	0.26	0.335
L5	0.121	0.041	0.624	0.159	0.055
Long2	0.032	0.514	0.025	0.392	0.037
LW1	0.152	0.114	0.045	0.068	0.62
LW11	0.184	0.054	0.211	0.495	0.057
LW13	0.12	0.172	0.499	0.113	0.097
LW16	0.083	0.753	0.034	0.087	0.044
LW18	0.036	0.048	0.243	0.594	0.079
LW20	0.25	0.082	0.21	0.198	0.26
LW29	0.056	0.064	0.094	0.392	0.393
LW34	0.375	0.096	0.042	0.447	0.04
LW36	0.049	0.041	0.64	0.153	0.117

LW37	0.069	0.029	0.367	0.507	0.028
LW38	0.179	0.186	0.037	0.516	0.082
LW6	0.166	0.478	0.083	0.226	0.047
LW7	0.748	0.057	0.09	0.061	0.045
LX	0.098	0.538	0.05	0.15	0.164
LY100	0.026	0.13	0.16	0.581	0.103
LY14	0.872	0.03	0.022	0.059	0.016
LY2	0.101	0.511	0.037	0.079	0.271
LY22	0.078	0.447	0.104	0.266	0.105
LY30	0.059	0.126	0.457	0.053	0.305
LY31	0.54	0.056	0.021	0.179	0.204
LY33	0.346	0.123	0.181	0.122	0.228
LY34	0.729	0.049	0.066	0.043	0.113
LY35	0.212	0.361	0.218	0.114	0.095
LY36	0.159	0.267	0.4	0.057	0.117
LY41	0.438	0.107	0.095	0.128	0.232
LY42	0.279	0.029	0.25	0.026	0.415
LY51	0.842	0.051	0.023	0.024	0.06
LY54	0.881	0.022	0.023	0.052	0.021
LY58	0.015	0.021	0.852	0.015	0.097
LY59	0.05	0.101	0.305	0.092	0.452
LY62	0.034	0.014	0.876	0.019	0.057
LY63	0.153	0.074	0.064	0.06	0.649
LY64	0.175	0.058	0.053	0.033	0.68
LY66	0.072	0.033	0.062	0.722	0.11
LY70	0.095	0.127	0.12	0.112	0.545
LY77	0.784	0.04	0.047	0.082	0.046
LY86	0.074	0.076	0.061	0.391	0.398
LY90	0.184	0.111	0.245	0.098	0.362
LY93	0.245	0.177	0.324	0.181	0.072
LY94	0.065	0.292	0.197	0.103	0.343
LY96	0.233	0.076	0.042	0.329	0.32
LY97	0.289	0.097	0.029	0.522	0.063
LY99	0.052	0.031	0.13	0.501	0.285
QD11	0.121	0.535	0.026	0.241	0.077
QD15	0.036	0.151	0.028	0.568	0.216
QD2	0.033	0.175	0.254	0.47	0.068
QD21	0.201	0.035	0.124	0.618	0.022
QD33	0.058	0.052	0.44	0.338	0.113
QD35	0.233	0.06	0.117	0.509	0.08
QD41	0.194	0.035	0.036	0.679	0.056
QD44	0.085	0.044	0.06	0.293	0.518
QD48	0.069	0.044	0.14	0.704	0.043
QD52	0.13	0.263	0.269	0.051	0.286

QD61	0.158	0.217	0.088	0.201	0.337
QD70	0.095	0.443	0.035	0.351	0.076
QD71	0.083	0.539	0.039	0.208	0.131
SJM	0.214	0.162	0.2	0.221	0.204
SS	0.231	0.086	0.054	0.519	0.11
TA1	0.163	0.027	0.206	0.048	0.556
TA10	0.292	0.145	0.142	0.216	0.205
TA11	0.028	0.283	0.025	0.04	0.625
TA12	0.442	0.027	0.449	0.03	0.052
TA13	0.025	0.022	0.024	0.024	0.906
TA14	0.024	0.022	0.023	0.025	0.906
TA15	0.046	0.107	0.093	0.335	0.42
TA16	0.025	0.125	0.018	0.027	0.804
TA17	0.062	0.053	0.023	0.845	0.017
TA18	0.033	0.015	0.081	0.025	0.845
TA19	0.043	0.163	0.171	0.343	0.281
TA2	0.666	0.024	0.14	0.045	0.123
TA20	0.052	0.05	0.772	0.048	0.078
TA21	0.057	0.266	0.182	0.12	0.375
TA22	0.183	0.075	0.129	0.095	0.519
TA23	0.274	0.09	0.268	0.292	0.077
TA24	0.064	0.057	0.141	0.045	0.693
TA33	0.37	0.032	0.289	0.035	0.274
TA4	0.035	0.138	0.063	0.056	0.708
TA5	0.023	0.107	0.822	0.021	0.027
TA6	0.649	0.044	0.073	0.139	0.095
TA9	0.631	0.133	0.073	0.113	0.05
WF100	0.056	0.043	0.097	0.05	0.753
WF103	0.045	0.089	0.357	0.117	0.392
WF105	0.31	0.059	0.239	0.305	0.087
WF106	0.158	0.424	0.038	0.145	0.235
WF108	0.457	0.129	0.075	0.131	0.209
WF110	0.028	0.297	0.037	0.024	0.614
WF115	0.126	0.077	0.191	0.149	0.457
WF119	0.019	0.065	0.018	0.842	0.056
WF120	0.064	0.041	0.126	0.514	0.254
WF124	0.052	0.038	0.617	0.24	0.053
WF15	0.031	0.045	0.081	0.453	0.39
WF17	0.065	0.038	0.041	0.83	0.026
WF18	0.044	0.099	0.101	0.6	0.156
WF19	0.375	0.164	0.037	0.291	0.133
WF20	0.131	0.011	0.749	0.039	0.07
WF22	0.025	0.117	0.06	0.367	0.43
WF25	0.075	0.038	0.11	0.078	0.698

WF27	0.023	0.019	0.017	0.907	0.034
WF29	0.068	0.062	0.082	0.225	0.563
WF32	0.021	0.043	0.018	0.889	0.029
WF40	0.34	0.048	0.061	0.486	0.065
WF45	0.018	0.032	0.026	0.049	0.874
WF49	0.084	0.109	0.162	0.584	0.061
WF5	0.403	0.027	0.328	0.198	0.044
WF50	0.017	0.032	0.86	0.057	0.033
WF52	0.037	0.019	0.035	0.856	0.053
WF53	0.198	0.222	0.029	0.397	0.153
WF56	0.071	0.428	0.029	0.451	0.022
WF60	0.02	0.753	0.046	0.124	0.057
WF62	0.043	0.053	0.035	0.054	0.815
WF65	0.02	0.049	0.022	0.02	0.89
WF69	0.864	0.053	0.031	0.027	0.025
WF70	0.028	0.113	0.027	0.045	0.787
WF77	0.038	0.865	0.015	0.05	0.032
WF78	0.115	0.119	0.044	0.308	0.414
WF80	0.083	0.472	0.061	0.3	0.083
WF85	0.02	0.076	0.113	0.029	0.761
WF90	0.016	0.015	0.909	0.035	0.024
WF93	0.127	0.016	0.02	0.787	0.049
WF94	0.184	0.086	0.081	0.616	0.034
WF95	0.022	0.318	0.044	0.055	0.561
WF97	0.114	0.042	0.302	0.435	0.107
WY	0.148	0.048	0.114	0.129	0.562
Y1	0.882	0.066	0.017	0.023	0.012
Y10	0.07	0.082	0.062	0.154	0.631
Y12	0.925	0.016	0.019	0.026	0.014
Y13	0.031	0.043	0.026	0.04	0.859
Y14	0.046	0.799	0.03	0.032	0.094
Y15	0.348	0.042	0.078	0.333	0.198
Y16	0.828	0.028	0.059	0.049	0.036
Y17	0.081	0.342	0.065	0.062	0.45
Y2	0.095	0.166	0.082	0.279	0.378
Y3	0.77	0.027	0.064	0.083	0.057
Y4	0.195	0.252	0.058	0.148	0.347
Y5	0.86	0.043	0.017	0.043	0.036
Y6	0.055	0.527	0.146	0.044	0.228
Y7	0.609	0.188	0.071	0.059	0.072
Y8	0.351	0.039	0.042	0.049	0.519
Y9	0.887	0.031	0.024	0.029	0.029
YT10	0.115	0.032	0.094	0.732	0.027
YT106	0.026	0.051	0.046	0.038	0.839

YT123	0.091	0.759	0.037	0.054	0.059
YT127	0.145	0.017	0.165	0.428	0.245
YT13	0.025	0.015	0.91	0.033	0.018
YT16	0.1	0.063	0.647	0.097	0.092
YT23	0.076	0.093	0.382	0.359	0.09
YT27	0.074	0.685	0.025	0.08	0.135
YT4	0.21	0.041	0.135	0.042	0.572
YT42	0.122	0.048	0.206	0.551	0.073
YT47	0.038	0.123	0.235	0.457	0.147
YT48	0.289	0.046	0.129	0.167	0.369
YT57	0.133	0.029	0.069	0.696	0.073
YT58	0.082	0.04	0.502	0.312	0.065
YT60	0.014	0.023	0.877	0.045	0.04
YT66	0.251	0.186	0.136	0.34	0.087
YT68	0.176	0.026	0.025	0.725	0.049
YT69	0.033	0.042	0.764	0.099	0.062
YT7	0.062	0.032	0.276	0.032	0.598
YT70	0.118	0.034	0.086	0.716	0.046
YT73	0.078	0.049	0.07	0.091	0.712
YT74	0.045	0.098	0.286	0.527	0.045
YT81	0.102	0.052	0.106	0.463	0.277
YT82	0.182	0.063	0.35	0.345	0.061
YT85	0.355	0.107	0.052	0.211	0.275
YT88	0.022	0.058	0.325	0.456	0.139
YT89	0.066	0.06	0.133	0.17	0.572
YT90	0.195	0.16	0.129	0.449	0.067
ZB1	0.03	0.019	0.018	0.839	0.093
ZB10	0.018	0.042	0.643	0.186	0.111
ZB11	0.023	0.12	0.049	0.081	0.727
ZB12	0.03	0.24	0.032	0.029	0.67
ZB13	0.401	0.047	0.255	0.234	0.063
ZB15	0.027	0.061	0.086	0.581	0.244
ZB17	0.145	0.068	0.076	0.116	0.595
ZB19	0.056	0.09	0.041	0.77	0.042
ZB21	0.051	0.059	0.029	0.245	0.616
ZB22	0.033	0.038	0.792	0.043	0.095
ZB25	0.124	0.051	0.557	0.074	0.193
ZB26	0.031	0.539	0.027	0.169	0.233
ZB27	0.131	0.177	0.238	0.24	0.213
ZB3	0.027	0.02	0.02	0.84	0.093
ZB31	0.063	0.089	0.133	0.637	0.078
ZB33	0.366	0.151	0.064	0.278	0.14
ZB5	0.285	0.534	0.03	0.08	0.071
ZB7	0.336	0.129	0.07	0.291	0.174

	ZB70	0.201	0.408	0.026	0.328	0.037
	ZB9	0.025	0.849	0.016	0.077	0.034
	ZZ10	0.153	0.637	0.032	0.117	0.061
	ZZ11	0.91	0.015	0.035	0.016	0.024
	ZZ12	0.61	0.033	0.104	0.116	0.136
	ZZ21	0.9	0.024	0.022	0.027	0.027
	ZZ22	0.536	0.103	0.114	0.114	0.133
	ZZ27	0.339	0.538	0.037	0.043	0.043
	ZZ28	0.707	0.058	0.171	0.041	0.022
	ZZ36	0.857	0.036	0.024	0.064	0.02
	ZZ39	0.704	0.033	0.149	0.062	0.052
	ZZ45	0.747	0.027	0.174	0.03	0.023
	ZZ46	0.889	0.029	0.022	0.021	0.039
	ZZ52	0.058	0.055	0.062	0.081	0.743
	ZZ56	0.59	0.027	0.224	0.113	0.046
	ZZ57	0.557	0.176	0.035	0.149	0.083
	ZZ71	0.767	0.039	0.022	0.057	0.115
	ZZ72	0.128	0.305	0.184	0.256	0.127
	ZZ76	0.525	0.209	0.044	0.193	0.028
	ZZ9	0.825	0.048	0.056	0.05	0.021
HB	HB13	0.066	0.582	0.058	0.057	0.237
	HB18	0.396	0.122	0.103	0.151	0.228
	HB2	0.039	0.211	0.424	0.267	0.059
	HB23	0.848	0.066	0.025	0.048	0.014
	HB25	0.038	0.21	0.099	0.044	0.609
	HB28	0.076	0.62	0.185	0.098	0.022
	HB29	0.269	0.048	0.042	0.596	0.045
	HB3	0.134	0.239	0.324	0.239	0.064
	HB30	0.203	0.066	0.066	0.607	0.058
	HB31	0.252	0.063	0.131	0.508	0.046
	HB38	0.042	0.748	0.105	0.025	0.08
	HB42	0.046	0.798	0.049	0.075	0.033
	HB46	0.027	0.842	0.064	0.035	0.032
	HB51	0.142	0.243	0.201	0.354	0.06
	HB55	0.067	0.111	0.682	0.126	0.014
	HB7	0.039	0.072	0.104	0.748	0.038
SX	SX11	0.024	0.868	0.044	0.027	0.038
	SX17	0.071	0.822	0.033	0.027	0.048
	SX22	0.015	0.883	0.019	0.026	0.056
	SX25	0.29	0.619	0.024	0.04	0.028
	SX26	0.02	0.831	0.068	0.043	0.039
	SX27	0.031	0.861	0.062	0.027	0.019
	SX28	0.034	0.733	0.043	0.14	0.049

SX29	0.021	0.926	0.014	0.022	0.017
SX31	0.02	0.907	0.019	0.015	0.039
SX33	0.058	0.213	0.362	0.261	0.106
SX34	0.016	0.776	0.034	0.034	0.14
SX35	0.017	0.919	0.017	0.019	0.029
SX38	0.022	0.925	0.014	0.022	0.017
SX43	0.019	0.906	0.033	0.03	0.013
SX5	0.138	0.501	0.241	0.095	0.026
SX53	0.024	0.862	0.065	0.03	0.019
SX59	0.095	0.822	0.024	0.036	0.023
SX7	0.016	0.832	0.027	0.039	0.085
SX72	0.013	0.936	0.023	0.011	0.018
SX73	0.04	0.552	0.057	0.331	0.02
SX78	0.087	0.692	0.05	0.078	0.094
SX81	0.025	0.903	0.022	0.027	0.022
SX84	0.023	0.889	0.02	0.023	0.045
SX88	0.132	0.69	0.028	0.047	0.103
SX92	0.038	0.73	0.179	0.025	0.027
SX94	0.085	0.744	0.058	0.063	0.051

Figure S1 Part of the primer screening images

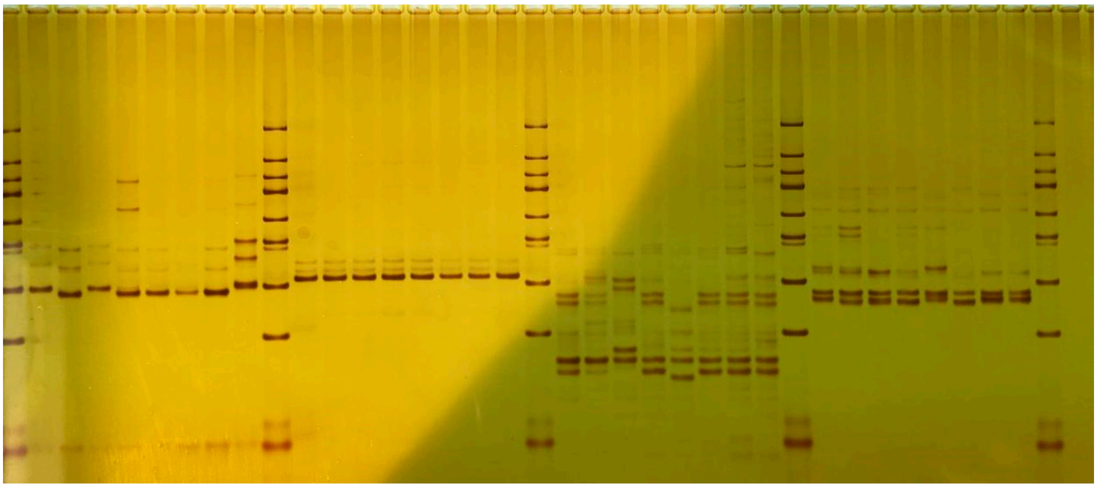

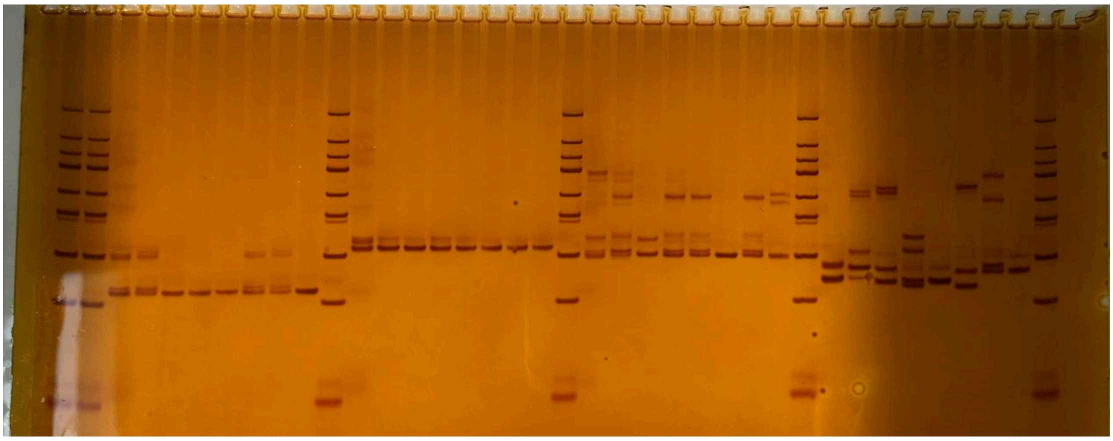

Supplement: Supplementary file 1 [file ijms-25-12776-s001.zip › ijms-3283015-supplementary.pdf]
